# An Observational Study of Sepsis in Takeo Province Cambodia: An in-depth examination of pathogens causing severe infections

**DOI:** 10.1371/journal.pntd.0008381

**Published:** 2020-08-17

**Authors:** Michelle Rozo, Kevin L. Schully, Casandra Philipson, Amitha Fitkariwala, Dararith Nhim, Tin Som, Darith Sieng, Bora Huot, Sokha Dul, Michael J. Gregory, Vireak Heang, Andrew Vaughn, Te Vantha, Angela M. Prouty, Chien-Chung Chao, Zhiwen Zhang, Tatyana Belinskaya, Logan J. Voegtly, Regina Z. Cer, Kimberly A. Bishop-Lilly, Chris Duplessis, James V. Lawler, Danielle V. Clark

**Affiliations:** 1 Austere environments Consortium for Enhanced Sepsis Outcomes (ACESO), Biological Defense Research Directorate, Naval Medical Research Center-Frederick, Ft. Detrick, Maryland, United States of America; 2 The Henry M Jackson Foundation for the Advancement of Military Medicine, Bethesda, Maryland, United States of America; 3 Genomics and Bioinformatics Department, Biological Defense Research Directorate, Naval Medical Research Center-Frederick, Fort Detrick, Maryland, United States of America; 4 Defense Threat Reduction Agency, Fort Belvoir, Virginia, United States of America; 5 Lucerent Clinical Solutions, Phnom Penh, Cambodia; 6 Chenda Polyclinic, Phnom Penh, Cambodia; 7 U.S. Naval Medical Research Unit TWO (NAMRU-2), Phnom Penh, Cambodia; 8 Takeo Provincial Referral Hospital, Takeo, Cambodia; 9 Viral and Rickettsial Diseases Department, Naval Medical Research Center-Silver Spring, Silver Spring, Maryland, United States of America; 10 Leidos, Reston, Virginia, United States of America; 11 Global Center for Health Security at Nebraska and Division of Infectious Disease, Department of Internal Medicine, University of Nebraska Medical Center, Omaha, NE, United States of America; Oxford University Clinical Research Unit, VIET NAM

## Abstract

The world’s most consequential pathogens occur in regions with the fewest diagnostic resources, leaving the true burden of these diseases largely under-represented. During a prospective observational study of sepsis in Takeo Province Cambodia, we enrolled 200 patients over an 18-month period. By coupling traditional diagnostic methods such as culture, serology, and PCR to Next Generation Sequencing (NGS) and advanced statistical analyses, we successfully identified a pathogenic cause in 46.5% of our cohort. In all, we detected 25 infectious agents in 93 patients, including severe threat pathogens such as *Burkholderia pseudomallei* and viral pathogens such as Dengue virus. Approximately half of our cohort remained undiagnosed; however, an independent panel of clinical adjudicators determined that 81% of those patients had infectious causes of their hospitalization, further underscoring the difficulty of diagnosing severe infections in resource-limited settings. We garnered greater insight as to the clinical features of severe infection in Cambodia through analysis of a robust set of clinical data.

## Author summary

We enrolled 200 severely ill patients in an observational study of sepsis in Takeo Province Cambodia. In an effort to provide an in-depth characterization of their illness, we combined standard clinical microbiology practices (culture, microscopy, etc) with serological analyses, newer molecular diagnostics, and next-generation sequencing to produce the most detailed characterization to date of sepsis and microbiological cause for southern Cambodia. Specifically, we were able to identify a causative pathogen in 46.5% of cases. A viral source was identified in 26 (13%) patients and bacterial source in 79 (39.5%) patients. In all, we detected 139 infections in 93 (46.5%) patients. Despite our best efforts though, 52.5% of the cohort remained undiagnosed, further underscoring the urgent need for new infectious disease diagnostics that can identify the cause of infections in near real time.

## Introduction

As a common pathway leading to severe disease and death from infection, sepsis is a leading cause of illness and mortality world-wide [1]. Although documented incidence rates of sepsis are rising, it is still likely underreported, especially in low-resource settings, due to diagnostic uncertainty [2]. Complicating matters, an international consensus panel recently updated its definition of sepsis (Sepsis-3), confining the label to patients with objective evidence of organ system dysfunction [2]. While this new definition may increase specificity and correctly identify patients with a higher risk of mortality relative to employment of the previous definition (Sepsis-1), it may lead to delayed recognition of patients who still ultimately exhibit poor outcome[3]. Improved diagnosis is imperative in sepsis to optimize treatment and prevent inappropriate interventions. Despite concerted effort however, we lack effective predictive biomarkers able to differentiate patients presenting with infectious versus non-infectious inflammatory syndromes. Thus, the diagnosis remains predominantly clinical and imprecise, contributing to antibiotic misuse and delays in appropriate antimicrobial treatment [4].

Culture-based identification remains the diagnostic gold standard for most bacterial pathogens, however the resources required to develop and maintain a high-quality clinical microbiology laboratory are formidable barriers in less-developed regions of the world. Organizations like the Diagnostic Microbiology Development Program (DMDP) (http://dmdp.org/) have been working to develop the capacity of diagnostic microbiology in Cambodia, but developing microbiology capacity from the ground up presents unique challenges [5]. By augmenting diagnostic microbiology, additional tools such as microscopy, rapid diagnostic tests (RDTs), molecular (RT PCR) tests, and serological assays can increase the diagnostic abilities of hospitals in resource-limited settings.

Next Generation Sequencing (NGS) potentially offers a comprehensive method for unbiased profiling of sepsis pathogens. While the use of sequencing for clinical diagnosis has long been suggested, recent advancements (i.e., increase in throughput, decrease in cost, etc.) have resulted in the integration of NGS into clinical and surveillance programs becoming increasingly more common [6–8]. Examples of direct sequencing of pathogens from clinical samples have been steadily increasing as the technology improves [9–11]. Bioinformatics expertise is paramount to sequence analysis, as NGS machines generate massive datasets that must be analyzed using specialized software on high performance computing (HPC) clusters. While cost and complexity will make NGS approaches untenable for resource-limited settings in the foreseeable future, application of this technique in select research settings can greatly enhance our understanding of sepsis in the developing world and can direct efforts to develop affordable diagnostic solutions.

The Austere environments Consortium for Enhanced Sepsis Outcomes (ACESO) is an international collaboration of researchers, led by the U.S. Naval Medical Research Center (NMRC), which executes a coordinated program of research aimed at improving early recognition, diagnosis, and effective treatment of severe infection from all causes in austere settings [12, 13]. In May of 2014, ACESO began an observational trial of sepsis at Takeo Provincial Hospital in Cambodia with the goal of identifying the common pathogens causing severe infections in Takeo Province Cambodia. Here, we describe a combination of standard clinical practices, laboratory diagnostics, and NGS utilized to identify the infectious causes of disease in a 200-patient cohort.

## Materials and methods

### Study site

Takeo is a predominantly rural province in southwest Cambodia with a majority of the workforce engaged in agriculture. Takeo Provincial Hospital (TPH) is a 250-bed public referral hospital for Takeo and surrounding provinces located in Krong Doun Kaev, Takeo Province, Cambodia [14]. The hospital admits over 1,000 patients per month for emergency, medical, obstetric, pediatric, and surgical care. While TPH offers a full range of hospital services, severely ill patients who require subspecialty or critical care are routinely transferred to hospitals in Phnom Penh. TPH possesses basic radiology and clinical laboratory services. In 2011, DMDP renovated the microbiology laboratory and continues to enhance microbiology capabilities within the TPH laboratory by providing training and supplies, such as blood culture bottles and selective and differential media for the isolation and identification of infectious bacteria [5].

### Ethics statement

Our study was designed as a prospective observational study of sepsis in Cambodia and was approved by the Naval Medical Research Center Institutional Review Board (NMRC.2013.0019), as well as the Cambodian National Ethics Committee for Health Research (NECHR). The study was conducted in compliance with all applicable Federal regulations governing the protection of human subjects. Treatment decisions were exclusively at the discretion of the hospital’s attending physicians. All patients, or their legally authorized representatives, provided written informed consent. Study subjects were assigned a volunteer identification number (VIN) upon enrollment, and were identified by VIN on case report forms and in the electronic database. All analyses were conducted on de-identified data.

### Participants

In May of 2014, ACESO began a prospective observational study of sepsis at TPH [14] and over the next 18 months enrolled 200 patients. Our study was implemented prior to the publication of Sepsis-3 guidelines [2] and thus patients were enrolled according to an earlier definition of sepsis [15]. Adult patients (≥18 years) admitted to TPH within the last 48 hours and who had a suspected infection (as judged by the attending physician) and met at least two of three clinical criteria (thermodysregulation defined as Temperature >38°C or <36°C, tachypnea defined as Respiratory rate >20/min and tachycardia defined as heart rate > 90 bpm) were considered for inclusion [15]. Exclusion criteria included the following: patients who presented with known active malignancy, known history of nephrotic syndrome or hepatic failure, known immunosuppressive conditions (not including HIV) or medications, including high-dose steroid usage (≥20mg/day), chemotherapy, or radiation treatment within the past 3 months. Patients with a history of organ transplant, hemodynamically significant gastrointestinal bleeding, anatomic or functional asplenia, and general anesthesia or surgery in the past week were also excluded, as were patients who were pregnant or up to 6 weeks post-partum, had a hemoglobin under 7 g/dL, or weighed less than 45kg (later amended to 35kg due to the small stature of the population). Finally, hospital physicians had the ability to defer enrollment for patients they deemed too ill to participate or with an immediately terminal comorbidity. All participants or their legally authorized representatives (LAR) provided written informed consent.

### Study-specific procedures

All clinical interventions were provided by and left to the discretion of the attending physicians and hospital medical teams, independent of study procedures. Following informed consent, study team members conducted a history and physical exam and used a standardized case-report form to record demographics, medical history, physical exam findings, and admitting diagnosis. If the patient was unable to provide demographic or symptom information, the data were collected from their LAR. At the time of enrollment, an HIV test (HIV ½ STAT-PAK, CHEMBIO diagnostic) was conducted and blood was aseptically collected from a single site for aerobic blood culture. Additional blood samples were also collected for analysis of peripheral venous blood gases with lactate (epoc, **Siemens Healthcare Diagnostics, Inc.**, Tarrytown, NY), complete blood counts (QBC Autoread Plus Dry Hematology System, Drucker Diagnostics, Port Matilda, PA) and clinical chemistries (Piccolo Xpress Chemistry Analyzer Abbott, Princeton, NJ). Finally, blood was drawn into tubes containing clot activators, anticoagulants (K_2_EDTA, BD, Franklin Lakes, NJ) and RNA stabilization matrix (PAXgene, PreAnalytix, Switzerland) for serology, targeted PCR, and RNA sequencing, respectively. Patients were followed throughout their treatment and a record review was conducted at discharge. Patients were requested to return for a follow up at 28 days where clinical samples, longitudinal symptoms and physical exam results were collected. When patients could not return in person, study team members attempted to conduct an interview with patients or LAR by telephone. Mortality in survivors discharged from the hospital was also evaluated at this time.

Hospital clinicians ordered additional microbiological testing for patient care when deemed appropriate. For example, additional blood cultures, cerebrospinal fluid (CSF), sputum (Giemsa stain, acid-fast stain), urine, and/or wound swab could be collected and tested per hospital protocol and results were recorded as part of the study case report form. Rapid diagnostic tests were also performed at the discretion of the attending physician and recorded in a similar manner for hepatitis B virus, hepatitis C virus, and malaria (ONE STEP HBsAg, ONE STEP Anti-HCV, ONE STEP Malaria HRP-II (P.f.) and pLDH (P.v.), respectively, Standard Diagnostics, Republic of Korea).

Pathogens were identified using a tiered approach detailed below. Briefly, direct detection of pathogens (culture, microscopy) or pathogen-associated molecules (nucleic acids, antigens) were given the highest level of confidence. Serological evidence of infection (i.e., seroconversion) was considered second and finally NGS-based detection of pathogens directly out of patient samples was used as a third-tier tool to support evidence gathered from traditional methods, and as a method for pathogen discovery.

### Record adjudication

Two independent physicians, board certified in internal medicine and/or infectious disease, reviewed all available hospital information to determine clinical syndrome, presence of infection, source of infection, pathogen, and degree of confidence in pathogen. When determinations from these two reviewers did not concur, a third independent reviewer, board certified in infectious diseases, reviewed the record as a tie-breaker. The committee used five categories to sort patients: “infectious”, “non-infectious”, “unknown”, “unknown–likely infectious”, and “unknown–likely non-infectious”. For pathogens identified by laboratory testing, clinical adjudicators provided judgment on clinical significance, and any identified organisms that were considered contaminants, colonizers, or unlikely causes of the present clinical syndrome were discounted. In a number of cases, more than one organism was identified in a patient where coincident infection could not be ruled out. In these cases, multiple pathogens were reported.

### Direct identification of etiological agents

Aseptic sample collection was performed by the nurse at respective wards of the hospital for patients enrolled in the study. Ten milliliters of blood was aseptically collected from a single site into culture bottles provided by DMDP and incubated at 35°C for seven days. At the discretion of the treating physician, a second culture bottle was drawn for some patients. Turbid culture bottles were Gram-stained and streaked onto appropriate selective and differential media (Ashdown’s, Chocolate, MacConkey) and standard biochemical characterizations were performed using the appropriate API strip (bioMérieux). Clinical isolates were transported to the Naval Medical Research Unit-2 in Phnom Penh for confirmatory testing and storage. There, antibiotic susceptibility testing (AST) was performed using a combination of the disk diffusion method and Etest (bioMérieux). The minimum inhibitory concentrations (MIC) were determined using guidelines from the Clinical and Laboratory Standards Institute (CLSI) [16]. Suspected *Burkholderia pseudomallei* isolates were identified and characterized as previously described [14] and subsequently destroyed in accordance with US DoD regulations. Additionally, the capsular polysaccharide of *B*. *pseudomallei* was detected in patient serum using the recently described *B*. *pseudomallei* iSTAT assay [17]. Antimicrobial Sensitivity Testing (AST) was performed on isolates as previously described [14]. Smears were prepared using quality sputum samples (i.e., <10 epithelial cells per low powered field) for Acid Fast Staining (Ziehl-Neelsen) and *Plasmodium* sp. microscopy (Giemsa stain). Regardless of quality, sputum was plated on Ashdown’s medium and quality sputum samples were plated on chocolate agar, MacConkey agar and sheep’s blood agar (SBA). Agar plates were incubated at 35°C.

### Quantitative PCR

DNA was extracted from 200 μL of serum from each patient collected at time zero (00h). DNA was extracted using the DNA mini extraction kit (Qiagen, Hilden, Germany) according to the manufacturer’s instructions. The DNA was then eluted in 200 μL of Tris EDTA (TE) buffer. DNA was either immediately used or stored at -20°C until molecular analysis. RNA was extracted from 140 μL of serum collected at time zero using the QIAamp viral RNA mini extraction kit (Qiagen, Hilden, Germany) according to the manufacturer’s instructions. RNA was then eluted in 60 μL of TE buffer. RNA was either immediately used or stored at -20°C until molecular analysis.

Serum DNA was tested using PCR with genus-specific primers and probes targeting specific sequences of *Haemophilus influenzae*, *Leptospira* sp., *Neisseria meningitidis*, *Salmonella typhi*, *Streptococcus pneumoniae*, *and Streptococcus suis* (Table 1). Real-time quantitative PCR was carried out according to the referenced protocols using a T100 Thermocycler (Bio-Rad, Hercules, CA, USA) for *Salmonella typhi* and Applied Biosystems 7500 Fast Real-Time PCR System for the others, and the TaqMan Universal PCR Master Mix (Thermo Fisher Scientific, MA, USA). For each assay, a DNA-free PCR mix was used as a negative control for PCR, and positive controls were as described in Table 1.

**Table 1:** Molecular Detection of Pathogens. Pathogens targeted for molecular detection are listed in the first column. The specific gene targets are listed in the next column followed by the source of the positive control utilized in the reaction. Finally, the references from which the assays were derived are listed in the last column.

Pathogen	Target	Positive Control	Reference
Chikungunya virus	E1 region of structural polyprotein gene	RNA isolated from serum of CHIKV patient	[18]
Dengue virus	Highly conserved region of N-terminal end in the NS5 gene	RNA isolated from serum of DENV patient	[19]
*Haemophilus influenzae*	*bexA* gene	DNA isolated from *H*. *influenzae* ATCC49247	[20]
Hantaviruses	S Segments of Dobrava, Hantaan, Puumala, and Seoul viruses	RNA isolated from serum of confirmed positive patient sample	[21]
*Leptospira* spp.	*lipL32* gene specific to pathogenic *Leptospira spp*.	Philippines strain *L*. *interrogans* strain AkiyamiB Serovar Hebdomadis	[22]
*Neisseria meningitidis*	*ctrA* gene	DNA isolated from *N*. *meningitidis* ATCC13090	[20]
*Salmonella typhi*	VI region of the flagellin gene	DNA isolated from *S*. *enterica* ssp enterica ser. typhimurium ATCC13311	[23]
*Streptococcus pneumoniae*	*ply* gene	DNA isolated from *S*. *pneumoniae* ATCC49619	[20]
*Streptococcus suis*	*cps2J* gene	DNA isolated from *S*. *suis* ATCC700796	[24]

Serum RNA samples were tested using primers and probes targeting Dobrava, Hantaan, Puumala, and Seoul viruses (Table 1), using the SuperScript III Platinum One-Step qRT-PCR Kit (Thermo Fisher Scientific, MA, USA). Additionally, RNA samples were tested using primers and probes targeting the N-terminal end of the NS5 gene of 51 flavivirus species and the QuantiTect SYBR Green RT-PCR Kit (Qiagen, Hilden, Germany), according to the manufacturer’s protocol. For each assay, a DNA-free PCR mix was used as a negative control for PCR, and positive controls were used as described in Table 1.

### Serologic testing

Through a combination of commercially available and laboratory-developed enzyme-linked immunosorbent assays, serological analyses were performed to detect a variety of disease agents that are common to Cambodia. Serological evidence of Chikungunya virus (CHIKV) and Dengue virus (DENV) infection was detected using human anti-CHIKV IgM and IgG ELISA (Abcam, USA), and DENV IgM and IgG Capture ELISA (Panbio, USA), respectively, which were performed according to the manufacturer’s instructions. Serological evidence of infection with *Orientia tsutsugamushi* was detected using an ELISA utilizing three recombinant proteins from four strains of *O*. *tsutsugamushi* (Karp, Kato, Gilliam and TA763) [25]. Spotted fever group rickettsia antibodies were detected by ELISA utilizing purified whole cell antigens from three species (*R*. *conorii*, *R*. *siberica* and *R*. *japonica*) [25], evidence of murine typhus was detected via antibodies to purified whole cell antigens from *R*. *typhi* using ELISA [26], Leptospirosis was also detected by ELISA using four recombinant proteins (LipL32, LipL41, LigA and LigB) from the pathogenic strain of *Leptospira interrogans* [27, 28], and ELISAs for evidence of *Coxiella burnetii* infection (causative agent of Q fever) employed a recombinant protein from a pathogenic strain (Henzerling) [29]. In all cases of laboratory-developed assays, cutoff values were derived from negative controls originating from neighboring Thailand that were previously confirmed to be antibody-negative as previously described [25, 29–31]. A four-fold increase in antibody titer (IgM, IgG) from sera collected at enrollment (acute) and 28 days later (convalescent) was considered positive. Confirmatory analyses of positive results were conducted by IFA as previously described (*O*. *tsutsugamushi)* [25] or according to the manufacturer’s instructions (*R*. *typhi*, Spotted Fever Group, Leptospirosis, and *Coxiella burnetii*, Fuller Laboratories, Fullerton, CA).

### Next generation sequencing

Total RNA was isolated from 2.5 ml of peripheral blood drawn directly into PAXgene RNA stabilization tubes (PreAnalytiX) using the PAXgene Blood miRNA Kit (Qiagen) according to the manufacturer’s instructions. RNA samples were evaluated using the Qubit 2.0 Fluorometer (Life Technologies, Carlsbad, CA, USA) and Agilent TapeStation (Agilent Technologies, Palo Alto, CA, USA). RNA library preparations and sequencing reactions were conducted at GENEWIZ, LLC. (South Plainfield, NJ, USA). Human rRNA and globin mRNA depletion was performed using Globin-Zero Gold rRNA Removal Kit (Illumina, San Diego, CA, USA). RNA sequencing library preparation was performed using the NEBNext Ultra RNA Library Prep Kit for Illumina by following the manufacturer’s recommendations (NEB, Ipswich, MA, USA). Briefly, enriched RNAs were fragmented for 15 minutes at 94°C. First strand and second strand cDNA were subsequently synthesized. cDNA fragments were end repaired and adenylated at 3’ends, and universal adapter was ligated to cDNA fragments, followed by index addition and library enrichment with limited cycle PCR. Sequencing libraries were validated using the Agilent TapeStation (Agilent Technologies, Palo Alto, CA, USA), and quantified by using Qubit 2.0 Fluorometer (Invitrogen, Carlsbad, CA) as well as by quantitative PCR (Applied Biosystems, Carlsbad, CA, USA). The resulting libraries were sequenced on the Illumina HiSeq 2500 to produce approximately 50 million reads in a 2×150 nucleotide paired end format. Sequences (post removal of incidental human-derived reads) are deposited in the NCBI Sequence Read Archive (SR) under project PRJNA408161.

### Bioinformatic analyses

EDGE Bioinformatics Software was used to classify the composition of microbial sequences derived from patients’ blood samples [32]. Briefly, raw sequence data (FASTQ files) were submitted to be processed using the EDGE pipeline as follows. Sequencing reads were filtered using high quality and trimming parameters, average quality score of 30. Human reads were subtracted from each sample using Bowtie2 (version 2.3.3); if a read aligned to the human reference genome (GRCh38) with ≥ 70% similarity via end-to-end alignment with Bowtie2, it was considered host contamination and was removed. Filtered, high-quality non-human reads were analyzed using the following read-based taxonomy classification tools embedded in EDGE software: Kraken Mini [33], BWA-mem mapping to a bacterial and viral subset of NCBI’s RefSeq [34], and GOTTCHA [35]. All EDGE parameters for taxonomy tools were set to default as described previously [36]. Patients were considered “positive” for microbe presence if all three taxonomy tools agreed. Coverage figures for reads aligning to the *O*. *tsutsugamushi* reference (NC_101793) were generated using CLCbio version 11 toolbox (*NGS Core Tool*, *Map Reads to Reference*) using default parameters. Heat maps were generated using R statistical programming.

### Statistical analysis

Clinical symptoms and clinical laboratory values were compared for patients with different infectious etiologies. Patients with apparent co-infections were counted as positive for multiple pathogens. Normality was evaluated graphically. Tests of statistical significance included chi-squared or Fisher’s exact test or the Wilcoxon rank sum test for non-normal data. All tests of statistical significance were two-tailed, and significance was defined as p<0.05. Statistical analyses were performed using Stata (version 14.1).

## Results

### Patient demographic and clinical information

**Fig 1:**
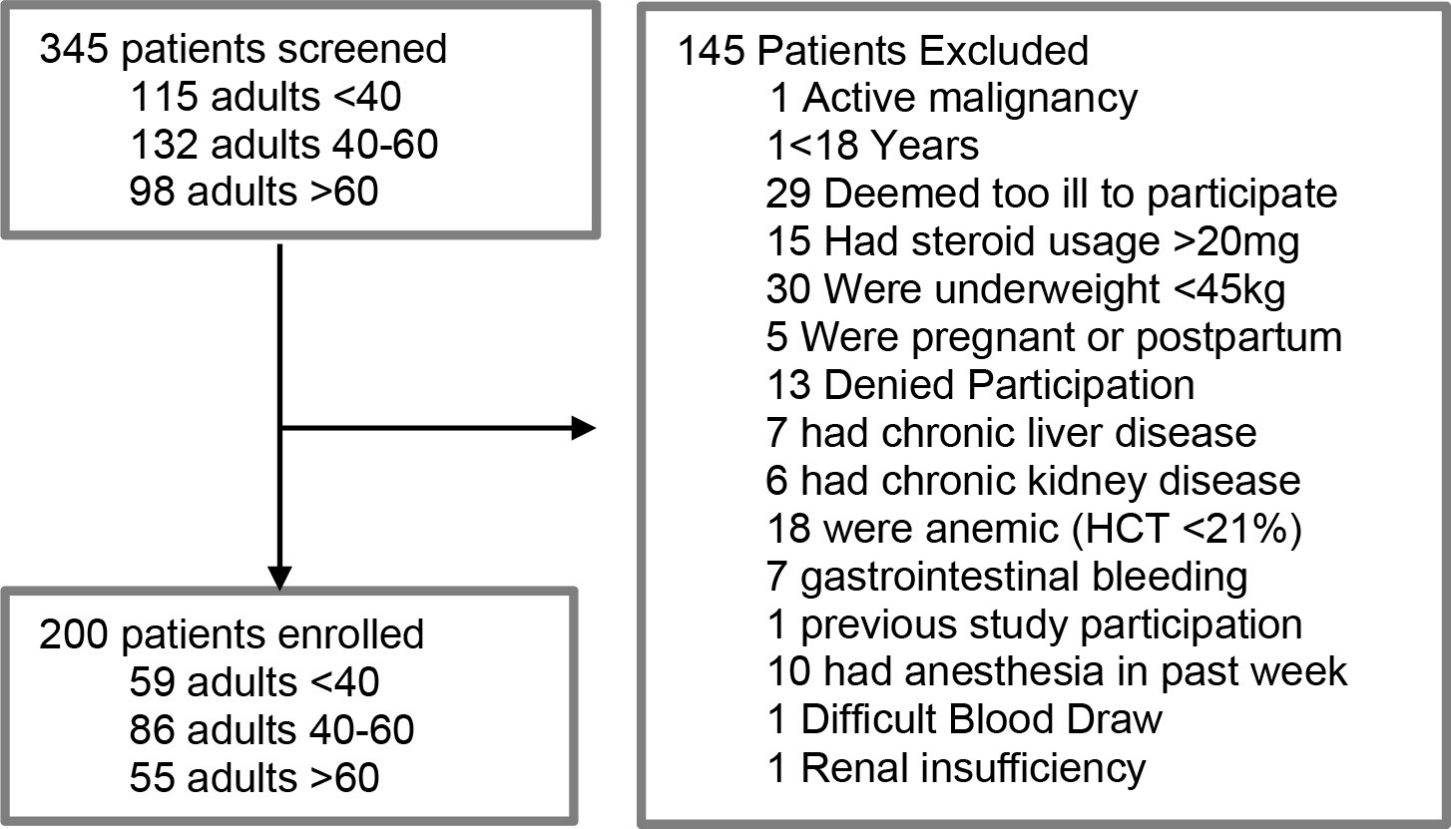
Schematic representation of study participants.

From May 30, 2014 to November 18, 2015 345 patients presenting to Takeo Provincial Hospital were screened for inclusion in the study [14]. One hundred forty-five patients were excluded from the study for specific reasons detailed in Fig 1. Ultimately, 200 patients were enrolled. Prior to enrollment, 46% (92/200) were receiving unpaid medical care in the home (i.e., family), 38.5% (77/200) were receiving paid professional care in the home, and the remainder (31/200, 15.5%) were receiving care from other clinics or hospitals. The median age was 50 (range 18–83) and the majority were male (n = 136, 68%) (Table 2). Thermodysregulation was present in 74.5% of patients, while 86.5% presented with elevated heart rate, and 99.5% with elevated respiratory rate. The median duration for hospital stay was 8 days (range 1–83), with the majority of patients (n = 173/200, 86.5%) at TPH for fewer than 16 days. Of the 182 patients for whom there was definitive 28-day data, 22 died, resulting in a 28-day fatality rate of 12.1%. At total of seven patients died prior to hospital discharge, with another six patients dying within a week of discharge. At 1-year follow-up, 42 of 122 (34%) patients had died. The median time to death was 26 days (range 1–342).

**Table 2:** Summary of study participants. Age, sex, days in the hospital and clinical outcome are summarized. The number (n) for each group is given with the percentage of that group in parentheses.

Age (in years)	n (%)
18–39	59 (29.5)
40–59	85 (42.5)
≥ 60	56 (28)
**Sex**	
Male	136 (68)
Female	64 (32)
**Days hospitalized**	
≤ 7 days	90 (45)
8 days to 15 days	83 (41.5)
≥ 16 days	24 (12)
Unknown	3 (1.5)
**28-day mortality**	
Survived	160 (80)
Died	22 (11)
Unknown	18 (9)

Patients originated from eight provinces in the southern and western parts of Cambodia; Takeo, Kampot, Kampong Speu, Kandal, Preah Sihanouk, Prey Veng, Koh Kong, and Battambang. The majority of patients were rice farmers (n = 109/200, 54.5%) or were not employed outside the home (n = 25/200, 12.5%) (S1 Table). Of those who reported their education level, 93/200 (46.5%) had received a primary education (Grade 1–6), while 52/200 (26%) had received no education. The majority of patients had no sick family members or friends (n = 160/200, 80%). Contact with cattle (n = 28/200, 14%), dogs (n = 7/200, 3.5%) and pigs (n = 7/200, 3.5%) were the most commonly described animal exposures. Of the patients enrolled, 115/200 (57.5%) were enrolled during the rainy season (May through October). The most common symptoms reported by enrolled patients included fatigue (n = 175/200, 87.5%), anorexia (n = 153/200, 76.5%), shortness of breath (n = 142/200, 71%), excessive sweating (n = 136/200, 68%), shaking/rigors (n = 133/200, 66.5%), headache (n = 132/200, 66%), cough (n = 123/200, 61.5%), and abdominal pain (n = 110/200, 55%) (S2 Table). Of the 174 patients who consented to an HIV test, 12/174 (6.9%) were positive.

Out of 200 patients, 93 patients (46.5%) received at least one microbiological diagnosis after all testing and adjudication was completed. Our retrospective clinical adjudication process identified a large number of the remaining patients as having suspected infections. Our panel adjudicated four patients to have a non-infectious cause of their illness. Of the 103 patients with no pathogen attributed to their illness, 92 (89%) were adjudicated to have infectious, or likely infectious, causes of their hospitalization. Using diagnostic assays available at the hospital microbiology lab (culture, microscopy) we were able to diagnose 38 patients. Thus 55 (59%) of our final 93 adjudicated microbiological diagnoses were made only with our more advanced serological and molecular assays or sequencing.

### Direct detection of pathogens, antigens and sequences reveals common causes of severe infections in Cambodia

A combination of culture, microscopy, and molecular techniques positively identified an infectious cause of disease in 49/200 patients (24.5%). With culture from all potential culture sites (blood, sputum, CSF, wound), 41 bacterial isolates and one yeast were obtained from 32/200 (16%) patients (Table 3). Sputum and blood culture (Table 3) produced similar numbers (18 and 20 respectively), followed by wound (n = 2) and cerebral spinal fluid (n = 1). Gram-negative bacteria (n = 33) dominated the culture results, with *B*. *pseudomallei* being the most commonly isolated pathogen (eight from blood, three from sputum), including seven patients previously described in detail [14]. Other notable gram-negative infections included *Klebsiella pneumoniae* (seven from sputum, one from blood), *Escherichia coli* (five from blood), *H*. *influenzae* (two from sputum), and *Pseudomonas aeruginosa* (two from sputum). Gram-positive bacteria were rare in this cohort; only three *Staphylococcus aureus* isolates (two from sputum, one from wound) and one *S*. *suis* isolate (CSF) were obtained. Also cultured, although at lower frequencies, were *Proteus mirabilis* (1), *S*. *typhi* (1), *Klebsiella ozaenae* (1), *Achromobacter xylosoxidans* (1), and unknown species of *Enterobacter* (1), *Acinetobacter* (1), and *Candida* (non-*albicans*).

**Table 3:** Pathogens identified and method of identification.

Pathogen	Total Number Identified	Methodology of Identification	Assay Specificity [reference]
*Achromobacter xylosoxidans*	1	Culture (Blood)	N/A
*Acinetobacter* sp.	1	Culture (Blood)	N/A
*Burkholderia pseudomallei*	23	Culture (8 Blood; 3 Sputum),	N/A
		I-STAT (12)	>93% [17]
*Candida non albicans*	1	Culture (Blood)	N/A
Chikungunya virus	9	Serology	>90% [product insert]
*Coxiella burnetti*	1	Serology	>96% [29]
Dengue virus	21	Serology (20)	100% [product insert]
		PCR/NGS	100% [19]
*Enterobacter* sp.	1	Culture (Sputum)	N/A
*Escherichia coli*	5	Culture (Blood)	N/A
*Haemophilus influenzae*	3	Culture (2, Sputum)	N/A
		PCR	100% [20]
Hepatitis B virus	1	RDT	>98% [41]
*Klebsiella ozaenae*	1	Culture (Sputum)	N/A
*Klebsiella pneumoniae*	8	Culture (7, Sputum; 1, Blood)	N/A
*Leptospira* sp.	10	Serology	86% [28]
Acid-fast bacilli	7	Microscopy	97% [40]
*Orientia tsutsugamushi*	16	Serology (9)	>87% [25]
		NGS (7)	unk
*Plasmodium* sp.	3	Microscopy (Giemsa Stain)	>85% [42]
*Proteus mirabilis*	1	Culture (Blood)	N/A
*Pseudomonas aeruginosa*	2	Culture (Sputum	N/A
*Rickettsia typhi*	4	Serology	unk
*Salmonella typhi*	1	Culture (Blood)	N/A
SFG rickettsioses	12	Serology (3 IgM Seroconversion)	58%
		Serology (9 IgG Seroconversion)	70%
*Staphylococcus aureus*	3	Culture (2, Sputum; 1, Wound)	N/A
*Streptococcus pneumoniae*	1	PCR	100% [20]
*Streptococcus suis*	1	Culture (CSF)	N/A

Abbreviations: NGS, Next Generation Sequencing; PCR, Polymerase Chain Reaction; RDT, Rapid Diagnostic Test; Unk, Unknown.

Antimicrobial sensitivity testing (AST) was conducted on bacterial isolates, and results consistent with previous studies in the geographical region were obtained [37–39]. For example, AST in *B*. *pseudomallei* isolates reliably demonstrated susceptibility to ceftazidime, amoxicillin/clavulanic acid and imipenem, and resistance to gentamicin, consistent with previously published reports [14]. Of gram-negative isolates, 75% (25/33 isolates), including 100% of the Enterobacteriaceae isolated from blood, demonstrated resistance to ciprofloxacin (S3 Table).

In addition to infectious etiologies identified by culture, microscopy identified seven patients with acid-fast bacilli (AFB) present in sputum smears. Because the presence of AFB in sputum is considered indicative of *Mycobacterium tuberculosis* infection, these patients were treated as tuberculosis (TB) patients [40]. Microscopy also identified *Plasmodium* species in three patients. Two patients were blood culture negative for bacteria but had serum DNA PCR positive for *H*. *influenzae* (n = 1) and *S*. *pneumoniae* (n = 1). Likewise, PCR analysis on RNA isolated from patient sera also positively identified infection with DENV in a single patient, although this patient was serum antibody negative. Hepatitis B virus (HBV) was confirmed in one patient through the detection of HBV surface antigens using the HBsAg Rapid Test, but this patient’s clinical picture was consistent with chronic HBV infection. Next generation diagnostics for melioidosis identified *B*. *pseudomallei* infections from an additional 12 culture negative patients. Eight of these patients produced melioidosis iSTAT results well over the previously established cutoff for 100% specificity and the remaining four were greater than 93% assay specificity [17]. These culture negative melioidosis patients bring the total number to 23 melioidosis cases.

**Fig 2:**
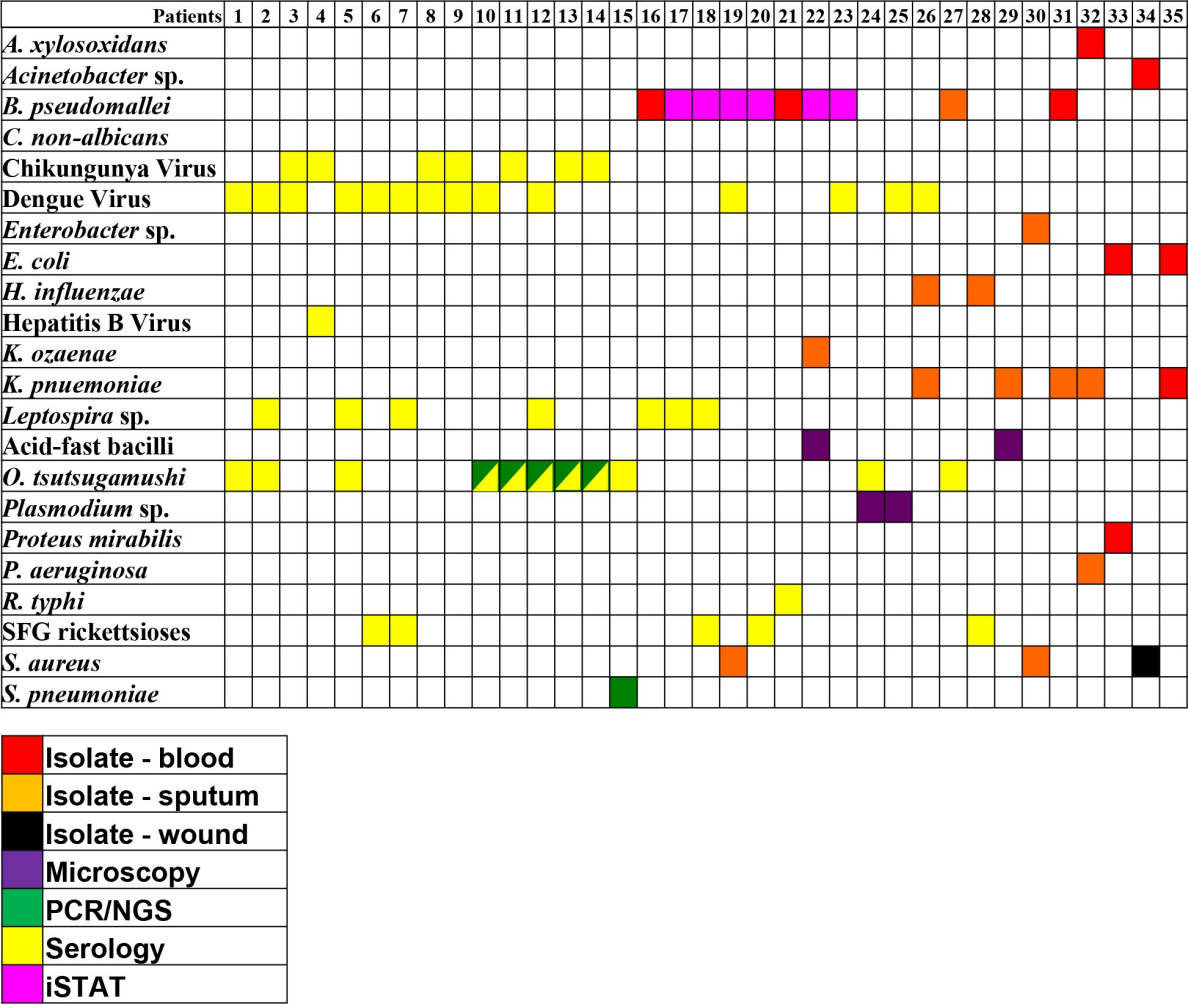
Coinfections identified in 200 patients. Potential co-infections indicated in 35 out of 200 patients. Each column, numbered 1–35 indicate the results from an individual patient. Colored boxes indicate a positive result from each patient for the pathogen listed in the first column, and the color of the box in the key below indicates the methodology used to detect evidence of that pathogen.

Culture positively identified several bacterial coinfections as well (Fig 2). Based upon clinical adjudication, our clinical reviewers could not identify a definitive single causative pathogen in these cases. In many subjects with multiple isolated pathogens, coinfection was plausible, as in Subject 29 (Fig 2) with apparent underlying lung disease and the identification of both AFB and *K*. *pneumoniae* in sputum. *E*. *coli* and *K*. *pneumoniae* were cultured from the blood of one patient; *E*. *coli* was co-cultured with *Proteus mirabilis* from another patient. *H*. *influenzae* and *K*. *pneumoniae* were each cultured from the sputum of a single patient; *K*. *pneumoniae* and *B*. *pseudomallei* were both isolated from another single patient. Two patients with acid-fast bacilli present in their sputum samples were also sputum culture positive for *K*. *pneumoniae* (n = 1) and *K*. *ozeane* (n = 1). Finally, one patient was culture positive for three bacteria: *K*. *pneumoniae* (sputum), *Achromabacter xylosoxidans* (blood), and *P*. *aeruginosa* (sputum).

### Community vs nosocomial pathogens

The overwhelming majority of organisms detected were consistent with community-acquired infections by clinical adjudication. Of the subjects with bacterial pathogens isolated, only seven were transferred from an outside hospital, and of these, only three were judged to have a clinical course prior to enrollment (including exposure at an outside hospital) so that nosocomial infection could not be ruled out. These subjects had two *Klebsiella* and one *Haemophilus* infection confirmed by culture.

### Serological analyses reveal viral, non-culturable bacterial, and potential co-infectious etiologies

In addition to direct detection of etiological agents from patient samples (i.e., culture, microscopy, PCR), immunological analyses were applied to detect serological evidence of infection for a variety of regionally important pathogens. Laboratory analyses of patient sera also detected seroconversion to viral and bacterial infections in 54 patients. Serological evidence of a viral infection was detected in 29 (14.5%) patients. Antibodies against DENV were most commonly identified (n = 20), followed by antibodies to CHIKV (n = 9); three patients were seropositive for both viruses.

In addition to viruses, seroconversion was detected for five bacterial agents not normally detected by standard culture methods. Specifically, seroconversion suggests infection by members of the spotted fever rickettsial group (n = 12), *O*. *tsutsugamushi* (n = 9), *Leptospira* spp. (n = 10), *R*. *typhi* (n = 4), and *Coxiella burnetti* (n = 1). Serology also identified numerous co-infections (Fig 2). Fourteen patients who demonstrated seroconversion were also positive for other pathogens identified by direct detection (culture, microscopy, PCR, iSTAT). Additionally, eleven patients produced multiple seropositive results.

### Next Generation Sequencing detects *Orientia tsutsugamushi* directly from clinical samples

**Fig 3:**
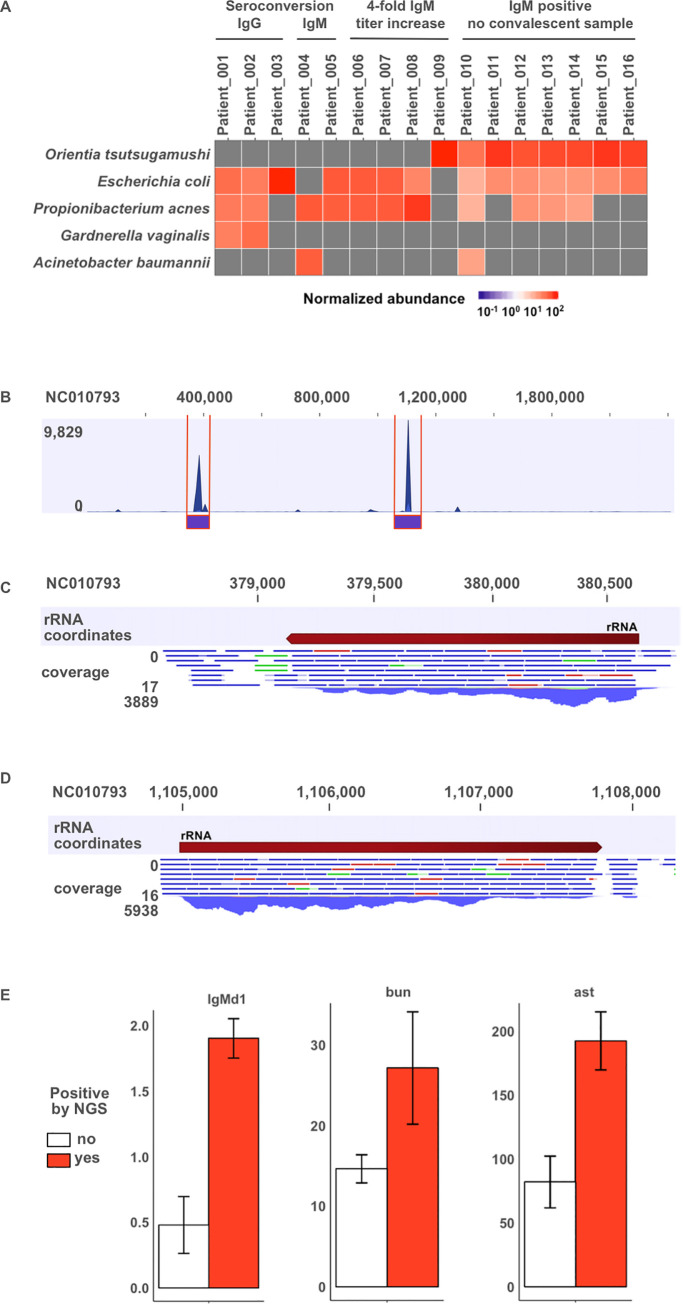
NGS-based microbial detection in blood. (A) Normalized read counts mapping to the top 5 microbes detected by GOTTCHA at the species level are presented for patients adjudicated with *Orientia tsutsugamushi* infection. Sequencing was performed on blood collected at initial hospital entry. Patient samples are ordered by method of adjudication: IgG seroconversion, IgM seroconversion, 4-fold IgM titer increase, or IgM positive with no convalescent sample. (B) Mapping *O*. *tsutsugamushi* positive reads to a reference genome (NCBI Accession: NC_010793) demonstrates high coverage in two regions representing 16S (C) and 23S (D) ribosomal RNA. (E) Acute IgM, Blood Urea Nitrogen (bun; mg/dL), and Alanine Aminotransferase (ast; U/L) measurements for *O*. *tsutsugamushi* are presented for patients grouped by NGS-based detection where group 1, colored white, indicates no detection by NGS, and group 2, colored red, indicates *O*. *tsutsugamushi* was detected by all taxonomy tools.

In order to detect potential etiological agents of sepsis in a broad and unbiased manner, RNAseq was performed on all acute whole blood samples. Microbes were identified using read-based taxonomy tools embedded in EDGE bioinformatics software [36, 43]. *O*. *tsutsugamushi* was one of the most common pathogens detected by sequencing. Laboratory analysis demonstrated that numerous patients were IgM positive for *O*. *tsutsugamushi* at the time of presentation, potentially indicating scrub typhus as the reason for their hospitalization. However, for patients who succumbed to infection or were lost to follow up, confirmation of seroconversion was not possible due to the lack of convalescent sera. Serology results were paired with RNAseq data to determine if NGS-based pathogen detection could assist etiological characterization of clinical samples. GOTTCHA, the most specific NGS-based classification tool of the tools used in this study, identified *O*. *tsutsugamushi* as a prevalent microbe in eight patient samples (Fig 3A), seven of which were from patients exhibiting elevated acute IgM titers but for whom 28-day follow-up samples were nonexistent. GOTTCHA results are supported by two additional taxonomy tools (S1 Fig, S2 Fig). To support *O*. *tsutsugamushi* positive calls, reads that aligned to the *O*. *tsutsugamushi* genome by BWA mapping to RefSeq were extracted and mapped to a reference genome. The majority of reads map to the 16S rRNA and 23S rRNA regions of the reference genome rather than throughout the coding regions of *O*. *tsutsugamushi* reference genomes (Fig 3B–3D; S3 Fig). These additional seven *O*. *tsutsugamushi* infections confirmed by NGS bring the total number of *O*. *tsutsugamushi* infections to sixteen.

### Clinical symptoms and laboratory values significantly associate with pathogen subgroups

We determined there were several statistically significant associations between pathogen and clinical symptoms or laboratory values at enrollment (Tables 4 and 5). Patients with evidence of *O*. *tsutsugamushi* infection were more likely to present with retro-orbital pain, blurred vision, sore throat, myalgia, abdominal pain, and rash and were more likely to have elevated AST and BUN, and lower total calcium, albumin, and total protein on laboratory analysis. Interestingly, the subgroup of *O tsutsugamushi* patients identified by NGS-based detection was associated with higher ALT and BUN compared with patients who were identified by serology (Fig 3E). Pharyngitis was also more common with DENV, occurring in 52%. Cough was universally present in patients identified with *K*. *pneumoniae* or AFB infection, and patients with AFB were also more likely to have elevated platelet count, low creatinine, low chloride, and hyponatremia whereas patients identified with *K*. *pneumoniae* had significantly lower AST levels than patients with other infections. Hyponatremia was statistically associated with *B*. *pseudomallei* infection as well, as was hyperglycemia. Patients testing positive for leptospirosis reported confusion (40%), and had significantly lower albumin levels. Lactate was significantly higher in patients testing positive for *E*. *coli*. Patients without an identified pathogen were significantly less likely to have presented with rigors, dizziness, and cough.

**Table 4:** Clinical symptoms associated with pathogens. Complaints of symptoms prior to hospitalization collected during enrollment. Bolded value with asterisk indicates the value is significantly different from patients without the indicated pathogen. Clinical symptoms were compared for patients with different infectious etiologies as described above. All tests of statistical significance were two-tailed, and significance was defined as p<0.05.

Symptom	None (N = 107)	*B*. *pseudomallei* (N = 23)	Dengue virus (N = 21)	*O*. *tsutsugamushi* (N = 16)	SFG (N = 12)	Leptospira spp. *(N = 10)*	*K*. *pneumoniae* (N = 8)	AFB (N = 7)	*E*. *coli* (N = 5)
*Fever*	95 (89%)	21 (91%)	20 (95%)	15 (93.75%)	11 (92%)	9 (90%)	6 (75%)	7 (100%)	5 (100%)
*Rigors*	60 (56%)*	19 (83%)	17 (81%)	13 (81.25%)	9 (75%)	8 (80%)	4 (50%)	7 (100%)	4 (80%)
*Sweat*	72 (67%)	17 (74%)	14 (67%)	13 (81.25%)	8 (67%)	8 (80%)	6 (75%)	3 (43%)	3 (60%)
*Dizzy*	37 (35%)*	13 (57%)	9 (43%)	8 (50%)	5 (42%)	5 (50%)	5 (63%)	4 (57%)	3 (60%)
*Headache*	68 (64%)	15 (65%)	14 (67%)	12 (75%)	8 (67%)	4 (40%)	6 (75%)	3 (43%)	3 (60%)
*Retro-orbital pain*	23 (22%)	7 (30%)	6 (29%)	8 (55%)*	2 (17%)	1 (10%)	3 (38%)	1 (14%)	2 (40%)
*Blurred vision*	41 (38%)	8 (35%)	10 (48%)	13 (81.25%)*	5 (42%)	5 (50%)	2 (25%)	3 (43%)	2 (40%)
*Hearing*	26 (24%)	4 (17%)	6 (29%)	6 (37.5%)	5 (42%)	2 (20%)	3 (38%)	2 (29%)	1 (20%)
*Confusion*	20 (19%)	3 (13%)	4 (19%)	4 (25%)	2 (17%)	4 (40%)*	0	0	0
*Stiff neck*	12 (11%)	1 (4%)	0	2 (12.5%)	0	0	1 (13%)	0	0
*Sore throat*	23 (22%)	6 (26%)	11 (52%)*	9 (56.25%)*	6 (50%)	4 (40%)	3 (38%)	3 (43%)	0
*Swollen glands*	2 (2%)	1 (4%)	0	0	0	0	0	0	0
*Shortness of breath*	71 (66%)	15 (65%)	15 (71%)	13 (81.25%)	8 (67%)	6 (60%)	8 (100%)	7 (100%)	4 (80%)
*Palpitation*	41 (38%)	11 (48%)	9 (43%)	9 (56.25%)	9 (75%)*	4 (40%)	4 (50%)	3 (43%)	4 (80%)
*Cough*	55 (51%)*	16 (70%)	14 (67%)	12 (75%)	10 (83%)	5 (50%)	8 (100%)*	7 (100%)*	4 (80%)
*Join pain*	34 (32%)	7 (30%)	10 (48%)	8 (50%)	6 (50%)	5 (50%)	1 (13%)	1 (14%)	2 (40%)
*Muscle soreness*	42 (39%)	10 (43%)	13 (62%)	16 (100%)*	7 (58%)	4 (40%)	3 (38%)	3 (43%)	1 (20%)
*Fatigue*	92 (86%)	19 (83%)	19 (90%)	14 (87.5%)	10 (83%)	8 (80%)	8 (100%)	7 (100%)	5 (100%)
*Anorexia*	80 (75%)	16 (70%)	16 (76%)	11 (68.75%)	10 (83%)	7 (70%)	8 (100%)	6 (86%)	5 (100%)
*Abdominal pain*	54 (50%)	11 (48%)	14 (67%)	14 (87.5%)*	8 (67%)	8 (80%)	4 (50%)	2 (29%)	4 (80%)
*Nausea*	41 (38%)	7 (30%)	7 (33%)	7 (43.75%)	4 (33%)	2 (20%)	1 (13%)	3 (43%)	1 (20%)
*Diarrhea*	19 (18%)	7 (30%)	4 (19%)	4 (25%)	4 (33%)	1 (10%)	0	1 (14%)	0
*Swelling*	7 (7%)	1 (4%)	3 (14%)	2 (12.5%)	1 (8%)	0	1 (13%)	0	1 (20%)
*Itching*	6 (6%)	1 (4%)	3 (14%)	3 (18.75%)	1 (8%)	0	0	0	0
*Rash*	4 (4%)	1 (4%)	2 (10%)	3 (18.75%)*	1 (8%)	0	0	0	0
*Skin lesions*	5 (5%)	1 (4%)	1 (5%)	0	0	0	1 (13%)	0	0
*Unusual bleeding*	0	0	0	0	0	0	0	0	0

**Table 5:** Summary of clinical laboratory results associated with pathogen groups. Clinical laboratory values were compared for patients with different infectious etiologies as described above. All tests of statistical significance were two-tailed, and significance was defined as p<0.05. Values are medians (IQR). Bolded value with asterisk indicates the value is significantly different from patients without the indicated pathogen. Note: WBC, White Blood Count; Hgb, Hemoglobin; Hct, Hematocrit; Na, Sodium; K, Potassium; Ca, Total Calcium; Cl, Chloride; BUN, Blood Urea Nitrogen; Cr, Creatinine; Glu, Glucose; Tbil, Bilirubin; Tp, Total Protein; ALB, Albumin; AST, Aspartate aminotransferase; ALT, Alanine Aminotransferase.

Clinical lab	None (N = 107)	*B*. *pseudomallei* (N = 23)	Dengue virus (N = 21)	*O*. *tsutsugamushi* (N = 16)	SFG (N = 12)	*Leptospira spp*. (N = 10)	*K*. *pneumoniae* (N = 8)	AFB (N = 7)	*E*. *coli* (N = 5)
WBC (cells/μl)	12.3×10^3^ (8–17.6)	14.3×10^3^ (9.7–18.5)	11×10^3^ (9–16)	11.1×10^3^ (8.8–12.8)	12.8×10^3^ (10.8–21.6)	10.7x10^3^ (9.8–12.1)	12.3×10^3^ (11.1–27)	11.5×10^3^ (9.2–13.9)	14.6×10^3^ (9.7–20.1)
Granulocyte %	83 (75–88)	84 (77–88)	80 (65–83)	77 (71–83)	81 (75–87)	83 (77–87)	81 (73–87)	76 (67–83)	85 (83–90)
Lymph/Mono Count (cells/μl)	2×10^3^ (1.5–2.8)	1.9×10^3^ (1.5–2.9)	2.2×10^3^ (1.7–3.1)	2.25×10^3^ (1.7–2.9)	2.5×10^3^ (1.9–3.9)	1.8x10^3^ (1.7–2.3)	2.7×10^3^ (2.2–3.5)	2.2×10^3^ (2.2–3)	2.7×10^3^ (1.4–3.1)
Hgb (g/dl)	12.4 (10.8–13.7)	11.9 (11–14.2)	12.7(11.8–13.9)	11.8 (10.8–12.9)	11.8 (10.9–12.3)	12 (9.6–12.8)	13.5 (11.7–14.7)	12.6 (11.4–14.6)	11.6 (10.7–11.9)
Hct (%)	38 (33–42)	36 (34–44)	39 (36–42)	36 (33–40)	37 (34–38)	38 (29–41)	40 (32–45)	42 (36–47)	32 (32–37)
Platelet (per μl)	2.87×10^5^ (2.02–4.24)	2.27×10^5^ (1.76–2.96)	2×10^5^ (1.52–2.49)	1.41×10^5^ (1.07–2.28)	3.17×10^5^ (1.82–3.66)	1.75x10^5^ (1.3–3)	2.79×10^5^ (1.50–7.53)	**6.54×10**^**5**^ (5.88–7.58)*	1.15×10^5^ (1.11–2.04)
Na (mmol/L)	135 (132–138)	131 (124–133)*	136 (133.5–139)	133 (129–138)	136.5 (135–138.5)	135 (133–137)	133 (129–134)	130 (129–133)*	135 (133–137)
K (mmol/L)	3.6 (3.1–4.2)	3.9 (3–4.5)	3.8 (3.4–4.4)	3.9 (3.3–4.3)	3.7 (3.2–4)	3.3 (2.4–3.5)	4.4 (3.2–5.3)	4.3 (3.2–4.4)	3.3 (2.6–3.8)
Ca (mg/dL)	2.2 (2.1–2.3)	2.1 (2–2.3)	2.1 (2.1–2.2)	2.1 (1.9–2.1)*	2.1 (2.1–2.3)	2.1 (2–2.2)	2.3 (2.1–2.4)	2.3 (2.1–2.5)	2.2 (2.2–2.2)
Cl (mmol/L)	97 (94–101)	93 (88–97)*	98 (96–101)	98 (95–101)	98 (98–102)	98 (93–102)	95 (89–99)	91 (90–93)*	101 (99–101)
BUN (mg/dL)	12 (9–15)	13 (10–17)	13 (11–20)	17 (11.5–21.5)*	10.5 (7–14)	12.5 (9–17)	13.5 (10.5–22)	11.5 (9–15)	14 (9–25)
Cr (mg/dL)	0.9 (0.7–1)	0.8 (0.7–1)	1 (0.9–1.3)	1 (0.9–1.5)	0.9 (0.8–1)	0.9 (0.7–1)	0.8 (0.5–0.9)	0.5 (0.5–0.6)*	1.1 (0.7–1.6)
Glu (mg/dL)	111 (93–135)	172 (110–330)*	110 (95–138)	99 (89.5–150)	109 (94–151)	131 (99–156)	122 (109–307)	111 (93–166)	99 (89–125)
Tbil (mg/dL)	0.8 (0.6–1.1)	0.9 (0.6–1.7)	0.9 (0.6–1.2)	.9 (0.65–3.3)	0.9 (0.6–2.5)	0.7 (0.6–0.9)	0.75 (0.6–1.1)	2.1 (0.6–3.6)	1 (0.5–4.9)
Tp (g/dL)	7.5 (7–8.1)	7.2 (6.6–7.7)	6.9 (6.2–7.8)	6.4 (5.6–7.4)*	7.1 (6.2–7.6)	6.5 (6.2–7.5)	7.8 (7.4–8.7)	7 (6.8–7.9)	7.8 (6.8–8.7)
Alb (g/dL)	3.1 (2.7–3.5)	2.5 (2.3–3.1)	2.8 (2.3–3.3)	2.4 (2–2.8)*	2.6 (2.4–3.1)	2.5 (2–3)*	2.6 (2.5–3.8)	2.5 (2.2–2.6)	3.1 (2.5–3.1)
AST (U/L)	55 (35–100)	58 (52–133)	67 (49–170)	154.5 (51.5–185.5)*	42 (32–155)	56 (45–76)	40 (32–56)*	100 (46–249)	56 (40–68)
ALT (U/L)	40 (25–74)	59 (32–95)	55 (38–85)	77.5 (45.5–116)	64 (30–95)	35.5 (19–52)	28 (25–43)	83 (44–159)	50.5 (32.5–73.5)
Lactate	21 (15.8–26.8)	23.2 (15.1–38.8)	20.4 (15.2–27.7)	19.5 (13.9–28.4)	19.9 (17–21.2)	21.8 (12.3–33.9)	25.2 (17.1–33.5)	20.7 (17.1–44.7)	26 (25.9–33.5)*

## Discussion

In this prospective study of 200 sepsis patients presenting to a provincial referral hospital in southeastern Cambodia, we detected 139 infections in 93 (46.5%) patients (Table 3). An infectious source was directly detected for 19 pathogens in 53 patients; serological evidence suggests infection with 7 pathogens in 54 patients and next generation sequencing verified the serology results in 8 patients. By coupling standard clinical microbiology practices with newer molecular diagnostics and next-generation sequencing, we were able to identify a causative pathogen in 46.5% of cases. A viral source was identified in 26 (13%) patients and bacterial source in 79 (39.5%) patients.

In this study we demonstrate that NGS-based detection enabled a diagnosis for some patients who were either culture negative, lacked convalescent serum specimens for paired serological analysis, or were otherwise undiagnosed by more traditional methods. By targeting RNA we focused the sequencing space on actively replicating transcripts indicative of replicating bacteria and, thus, active infection. *O*. *tsutsugamushi* was one of the most common pathogens detected by sequencing and was identified in seven patients where only the acute phase sample existed. We also detected DENV in the patient sample which yielded a positive PCR result (described above). The sequence data for DENV was so robust that 99.85% of the genome was sequenced, enabling detailed identification as DENV1 Strain 00407/95 accession JN638344.

To further investigate the utility of NGS-aided diagnostics for sepsis, *O*. *tsutsugamushi-*positive patient data were evaluated in detail. In the present cohort, a combination of serological analyses and NGS identified a total of sixteen *O*. *tsutsugamushi-*patients. Acute and convalescent blood draws led to confirmed serological diagnosis of *O*. *tsutsugamushi* for nine patients. Sequencing identified *O*. *tsutsugamushi* as the most prevalent microbe represented in blood RNA from seven additional study participants. All seven NGS-positive patients had high IgM titers in acute samples but lacked convalescent samples to complete serological analysis. In one case, a patient identified by serology with a four-fold increase in titer was confirmed by the detection of *O*. *tsutsugamushi* sequences by NGS. Presumably, detection of pathogen sequences is indicative of higher bacterial burden. Consistent with this hypothesis, patients with pathogen sequences detected by NGS were associated with more severe disease presentations as determined by clinical laboratory values (Tables 4 and 5; Fig 3E). Taken together, the results suggest that adopting a sequence-based detection method, global or targeted, in combination with traditional point-of-care blood panels could improve adjudication and time-to-diagnosis in future sepsis surveillance studies.

One distinction of our study is the depth and breadth of clinical data collected from the patient cohort over the time course of the study. These data allow a much richer appreciation of the clinical features of severe infection in Cambodia and allowed us to investigate specific associations with particular pathogens. For example, data from our 200-patient cohort (that included 11 culture-confirmed *B*. *pseudomallei* infections) is consistent with our previous observation that hyponatremia appeared to be disproportionately present in the first seven melioidosis cases [14]. Although hyponatremia is frequently associated with legionellosis and more severe community acquired pneumonia, the pathophysiologic mechanism is poorly understood [44–46]. Our data are insufficient to allow correlation of sodium level with presence or severity of pneumonia or whether the association with *B*. *pseudomallei* infection is independent of pulmonary involvement, thus further study is warranted. Tuberculosis patients were the only others with a statistically significant hyponatremia. TB patients also had a marked thrombocytosis and low creatinine compared to other patients, likely representing a more chronic inflammatory and catabolic state. Finally, patients with evidence of *O*. *tsutsugamushi* infection appeared to have a cluster of signs and symptoms that were more strongly associated with *O*. *tsutsugamushi* infection than other etiologies. This constellation included retro-orbital pain, visual disturbance, sore throat, myalgia, abdominal pain, rash, and BUN and AST elevation. We did not find a statistical association between *O*. *tsutsugamushi* and thrombocytopenia, although this is commonly ascribed [47]. The predilection of scrub typhus to present with liver and kidney abnormalities is consistent with the known clinical syndrome. The hypocalcemia noted was total calcium levels and thus likely proportional to hypoalbuminemia in those subjects. While our sample size is insufficient to make statistically significant conclusions about the disease-diagnostic utility of clinical laboratory values, many of our observations are consistent with prior descriptions of pathogen-associated syndromes and have biological plausibility to support a true association. These associated symptoms and laboratory values, and particular combinations thereof, deserve further investigation to determine whether clinical diagnostic criteria could help to identify patients more likely to have a specific pathogen where antimicrobial selection may differ (e.g. doxycycline for *O*. *tsutsugamushi* or ceftazidime for *B*. *pseudomallei*) and where timely microbiologic diagnosis is routinely unavailable in resource-limited hospitals.

RNA sequencing contributed to the depth of our dataset and aided in retrospective diagnosis of patients who succumbed to their illness or failed to return after hospital discharge. More real-time sequencing methods could eventually provide enhanced diagnostic ability, however, foreseeable techniques for sequencing-aided diagnosis will suffer from a number of limitations that we probably encountered in our study. Blood levels of circulating pathogens (and thus pathogen nucleic acid sequences) differ throughout the course of illness. Patients who are ill may have a bacterial or viral burden under the limit of detection of nucleotide-based methods. The liberal availability and wide-spread use of antibiotics in the general community in Takeo Province may also suppress bacteremia and sensitivity of sequencing, and many of our patients had self-treated with antibiotics prior to hospital presentation. Furthermore, resources limitations including space, consistent power, reliable cold chain and staff training make wide-spread use of real-time sequencing in resource-limited settings unlikely in the foreseeable future. As molecular (and sequenced-based) diagnostic testing methods become more widely available, clinicians must continue to learn how these methods provide useful diagnostic information and where increased sensitivity may also detect clinically insignificant infection and co-infection. Ten of the sixteen patients diagnosed with *O*. *tsutsugamushi* were adjudicated as having probable or possible co-infections, and it is possible that hospital admittance was driven by illness caused by a different pathogen. Finally, *E*. *coli* sequences were ubiquitously present in the samples. Because *E*. *coli* DNA is a common contaminant of laboratory reagents [48], we suspect that widespread detection of *E*. *coli* is a byproduct of sample processing. Consistent with that hypothesis, none of these patients had positive blood cultures for *E*. *coli*.

One limitation of our study is our reliance on serological evidence of infection for certain pathogens, with its inherent difficulty in interpretation and potential for false positives. Coinfection was suggested by serologic assays in over 10% of our patients, and in some instances, the likelihood of true coinfection versus false-positive serology was impossible to discern. For instance, the association of positive leptospirosis serology with culture-confirmed *B*. *pseudomallei* infection may reflect a common water source as a well-characterized risk factor for both. To improve accuracy, we followed CDC diagnostic guidelines for serum-only diagnoses and utilized recombinant antigens with no known cross-reactivity to other organisms [25, 27, 29]. Because background seropositivity for many pathogens we tested (spotted fever group rickettsia and *Leptospira* spp., for example) is high in this area, we applied high cutoff values and rigorous standards for our serum-only diagnoses.

We observed a relatively low rate of positive blood cultures and very few gram-positive organisms. We believe this is due to self-medication through the purchase of over-the-counter antibiotics, which is common in the region [49–51]. In fact, 70% (140/200) reported use of antibacterial agents prior to enrollment in the study and only 26 of those (18.5%) were referred from another hospital. Additionally, we believe that *B*. *pseudomallei* is underrepresented in our cohort. Melioidosis is diagnosed with the greatest success utilizing a “head-to-toe” culture approach. In our cohort, sputum cultures were not available for the first seven months of the study, leaving blood as the only biospecimen cultured during that time period. Finally, this study was conducted at a relatively small referral hospital at a single location in Cambodia and should not be considered a comprehensive representation of sepsis-causing organisms throughout Cambodia.

In this study, we present the most detailed characterization to date of sepsis and microbiological cause for southern Cambodia. The clinical associations we found may have significant impact in developing clinical prediction tools that may more accurately direct early antimicrobial selection and clinical decision making. It is important to note that not only did the utilization of serological, molecular and sequencing data essentially double our diagnostic yield, but these techniques were able to detect a number of pathogens that would necessitate different antimicrobial selection from common empiric therapy. Clearly, the serological and sequencing platforms are currently unable to yield real-time diagnostic data that can guide clinical decision making for a sepsis cohort in this region, and despite a multi-pronged approach for adjudication, 52.5% of the cohort remained undiagnosed. These facts highlight the urgent need for new infectious disease diagnostics that can identify infections in near real time. Such tools are even more important in resource-limited environments such as Takeo, where empiric antimicrobial selection is necessarily restricted by cost. In our study, we detected *B*. *pseudomallei*, *O*. *tsutsugamushi*, *Rickettsia* spp., or *Coxiella burnetti* in 26% of patients–diagnoses that would significantly alter antimicrobial management, resulting in improved outcomes. In addition, we detected only viral pathogens in 13 (6.5%), knowledge which could prompt withholding of antibiotic therapy, improved antimicrobial stewardship, and lower risk of emerging antimicrobial resistance. More research is needed to continue our understanding of causes and manifestations of sepsis in Southeast Asia, develop improved clinical predictors of specific syndromes, and most importantly to develop new diagnostic tools to facilitate management in resource limited settings.

## Supporting information

S1 Checklist STROBE Checklist. (PDF)

S1 Fig NGS-based microbial detection in blood by KrakenMini Taxonomy. Normalized read counts mapping to top 20 microbes (species level) detected by KrakenMini for patients adjudicated with *O*. *tsutsugamushi* infection.
(TIF)

S2 Fig NGS-based microbial detection in blood by BWA Taxonomy. Normalized read counts mapping to top 20 microbes (species level) detected by BWA mapping to RefSeq for patients adjudicated with *O*. *tsutsugamushi* infection.
(TIF)

S3 Fig Read mapping to *Orientia tsutsugamushi* reference (NC_010793). Reads that mapped to *O*. *tsutsugamushi* according to BWA in EDGE were extracted and mapped to a reference genome using CLC and visually inspected. Two regions of depth represent the 16S and 23S ribosomal RNA for all samples. The Y axis represents the depth of coverage (DOC), X axis represents the *O*. *tsutsugamushi* reference genome (NC_010793).
(TIF)

S1 Table Demographics of enrolled patients. (DOCX)

S2 Table Baseline characteristics of enrolled patients. (DOCX)

S3 Table Summary of identified pathogens with methodology of identification. (DOCX)
