# Supplementary figures and images for: An Observational Study of Sepsis in Takeo Province Cambodia: An in-depth examination of pathogens causing severe infections
Source: PLoS Negl Trop Dis. 2020 Aug 17;14(8):e0008381. doi: 10.1371/journal.pntd.0008381 (PMC7430706; doi:10.1371/journal.pntd.0008381)

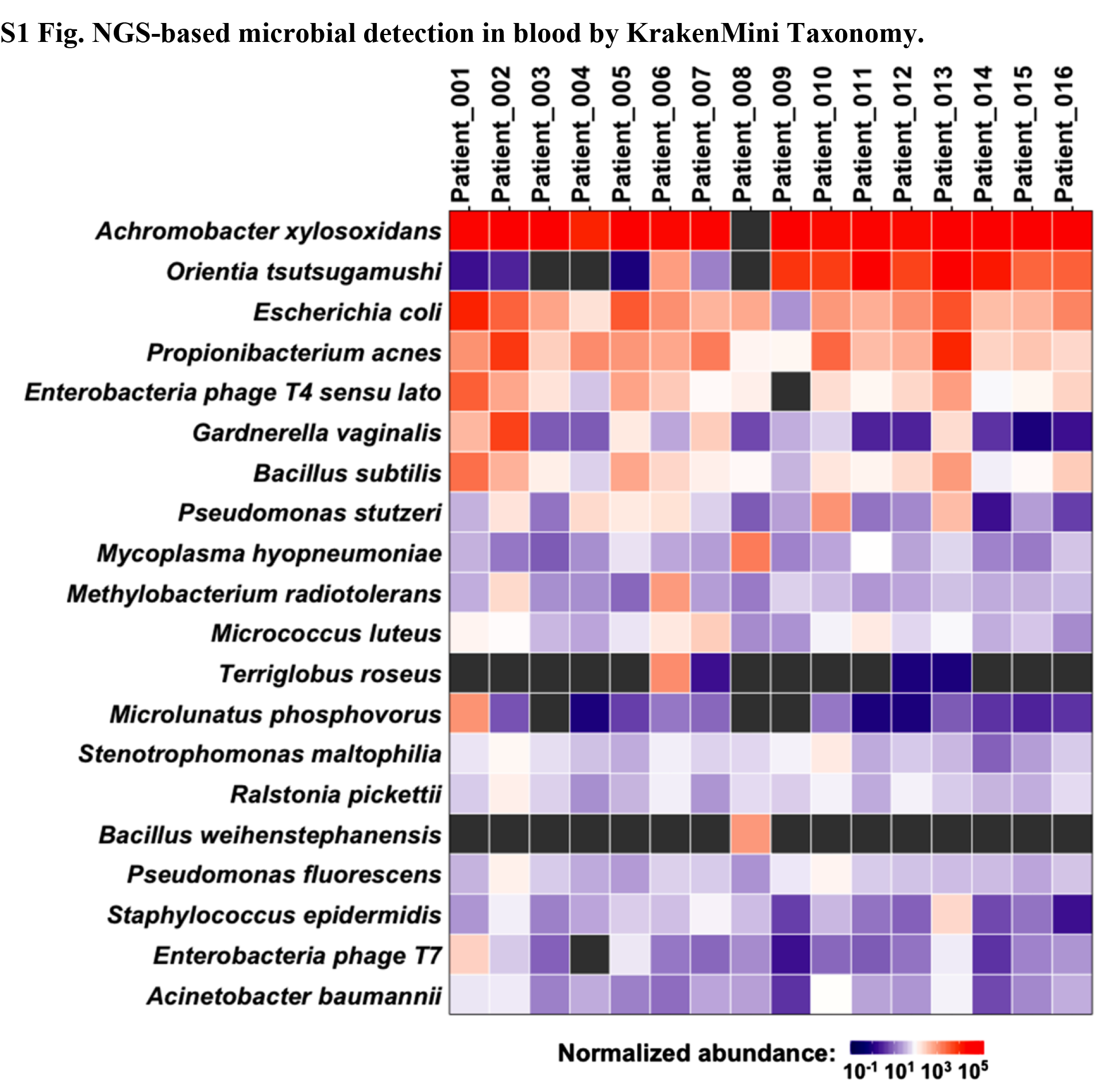

Supplement: S1 Fig — Normalized read counts mapping to top 20 microbes (species level) detected by KrakenMini for patients adjudicated with O. tsutsugamushi infection. (TIF) [file pntd.0008381.s002.tif]

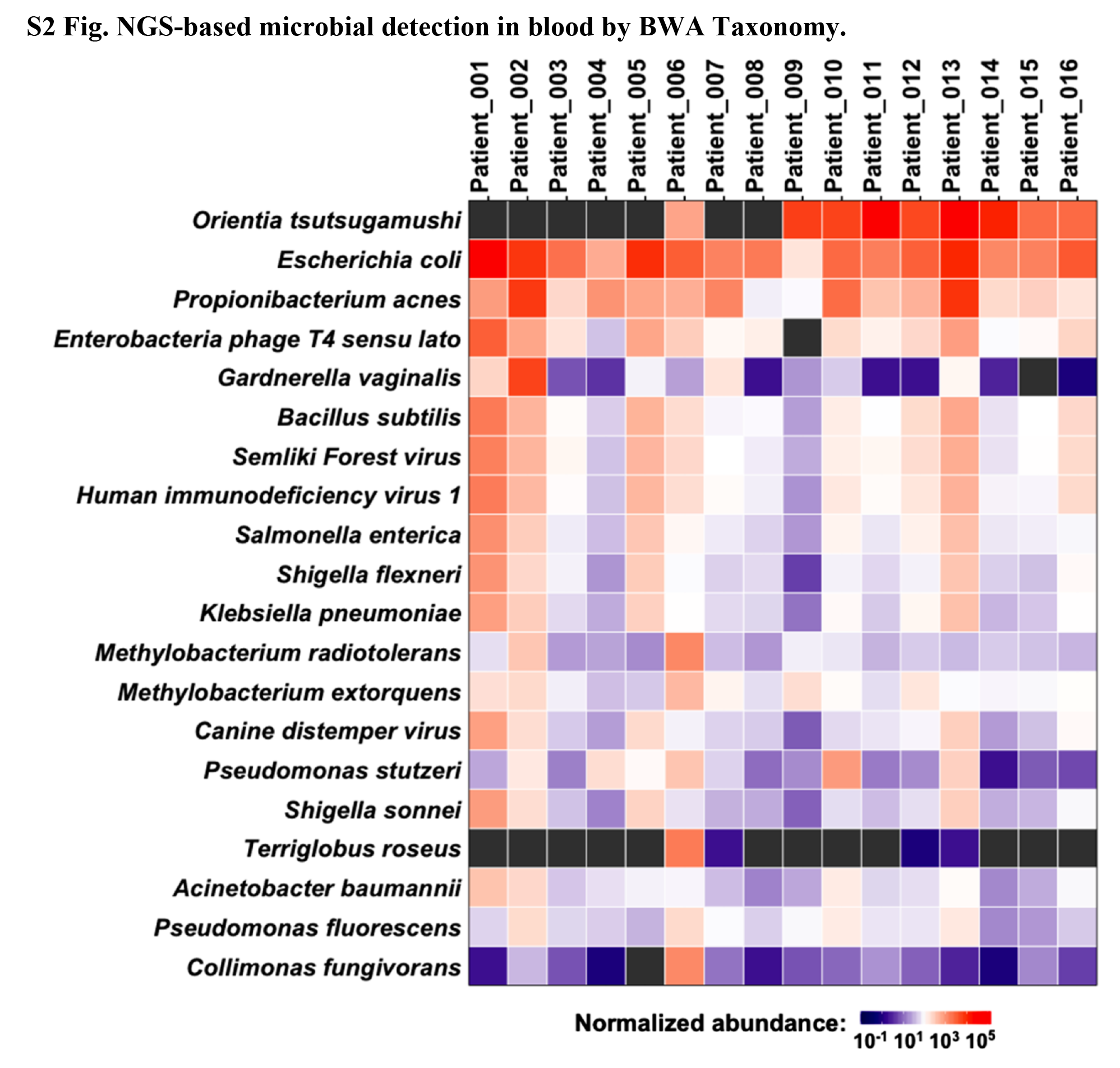

Supplement: S2 Fig — Normalized read counts mapping to top 20 microbes (species level) detected by BWA mapping to RefSeq for patients adjudicated with O. tsutsugamushi infection. (TIF) [file pntd.0008381.s003.tif]

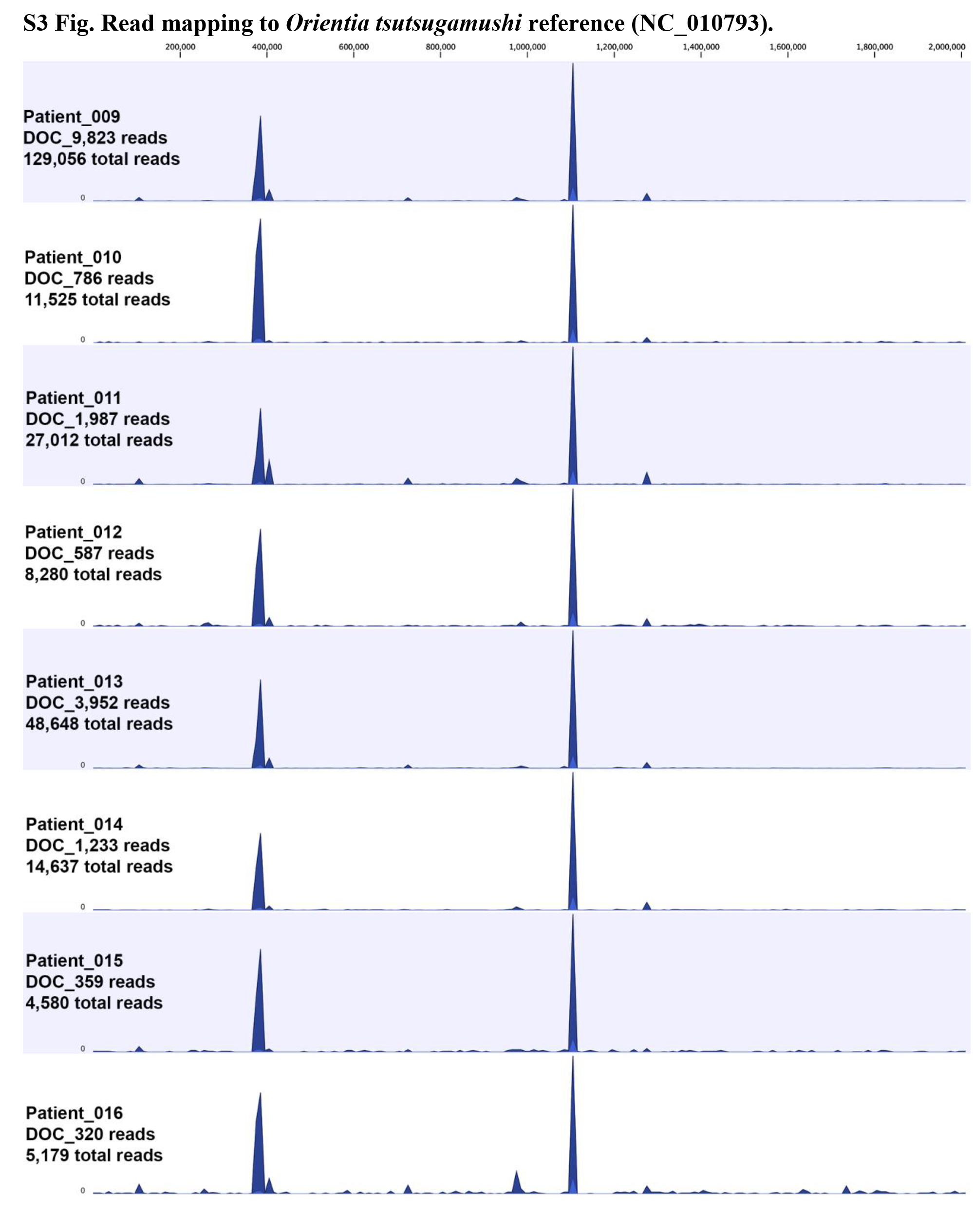

Supplement: S3 Fig — Reads that mapped to O. tsutsugamushi according to BWA in EDGE were extracted and mapped to a reference genome using CLC and visually inspected. Two regions of depth represent the 16S and 23S ribosomal RNA for all samples. The Y axis represents the depth of coverage (DOC), X axis represents the O. tsutsugamushi reference genome (NC_010793). (TIF) [file pntd.0008381.s004.tif]
