# Supplementary material for: An Observational Study of Sepsis in Takeo Province Cambodia: An in-depth examination of pathogens causing severe infections
Source: PLoS Negl Trop Dis. 2020 Aug 17;14(8):e0008381. doi: 10.1371/journal.pntd.0008381 (PMC7430706; doi:10.1371/journal.pntd.0008381)
Supplement: S1 Table — (DOCX) [file pntd.0008381.s005.docx]

**S1 Table. Demographics of enrolled patients**

| **Demographics** | |  | **N (%)** |
| --- | --- | --- | --- |
| Occupation | |  |  |
|  | Dry Farming |  | 12 (6) |
|  | Rice Farming |  | 109 (54.5) |
|  | Abattoir |  | 1 (0.50) |
|  | Factory |  | 7 (3.5) |
|  | Teaching |  | 4 (2) |
|  | Fishing |  | 2 (1) |
|  | Trade |  | 2 (1) |
|  | Laborer |  | 6 (3) |
|  | Home |  | 25 (12.5) |
|  | Other |  | 32 (16) |
| Education Level | |  |  |
|  | None |  | 52 (26) |
|  | Primary (Grade 1-6) |  | 93 (46.5) |
|  | Secondary (Grade 7-9) |  | 33 (16.5) |
|  | High School (Grade 10-12) |  | 16 (8) |
|  | Diploma (2 years) |  | 3 (1.5) |
|  | Bachelor (4 years) |  | 3 (1.5) |
|  | Advance Degree (Masters, MD, PHD) |  | 0 (0) |
| Animal Contact | |  |  |
|  | None |  | 140 (70) |
|  | Dogs |  | 7 (3.5) |
|  | Cats |  | 2 (1) |
|  | Cows |  | 28 (14) |
|  | Pigs |  | 7 (3.5) |
|  | Bats |  | 0 (0) |
|  | Monkeys |  | 0 (0) |
|  | Rodents |  | 0 (0) |
|  | Other |  | 29 (14.5) |
| Patient Contact with Sick Family/Friend | |  |  |
|  | Yes |  | 28 (14) |
|  | No |  | 172 (86) |
| Patients Enrolled Season | |  |  |
|  | Rainy Season (May - October) |  | 115 (57.5) |
|  | Dry Season |  | 85 (42.5) |
