# Supplementary material for: An Observational Study of Sepsis in Takeo Province Cambodia: An in-depth examination of pathogens causing severe infections
Source: PLoS Negl Trop Dis. 2020 Aug 17;14(8):e0008381. doi: 10.1371/journal.pntd.0008381 (PMC7430706; doi:10.1371/journal.pntd.0008381)
Supplement: S2 Table — (DOCX) [file pntd.0008381.s006.docx]

**S2 Table. Baseline characteristics of enrolled patients**

| **Symptom** | **N (%)** |
| --- | --- |
| Fever | 182 ( 91.0 ) |
| Shaking/Rigors | 133 ( 66.5 ) |
| Sweating | 136 ( 68.0 ) |
| Dizziness | 85 ( 42.5 ) |
| Headache | 132 ( 66.0 ) |
| Pain Behind Eyes | 49 ( 24.5 ) |
| Visual Blurring | 80 ( 40.0 ) |
| Hearing Problem | 54 ( 27.0 ) |
| Confusion | 30 ( 15.0 ) |
| Stiff Neck | 17 ( 8.5 ) |
| Sore Throat | 54 ( 27.0 ) |
| Swollen Glands | 3 ( 1.5 ) |
| Shortness of Breath | 142 ( 71.0 ) |
| Palpitations | 89 ( 44.5 ) |
| Cough | 123 ( 61.5 ) |
| Pain in Joints | 70 ( 35.0 ) |
| Muscle Soreness | 90 ( 45.0 ) |
| Fatigue | 175 ( 87.5 ) |
| Anorexia | 153 ( 76.5 ) |
| Abdominal Pain | 110 ( 55.0 ) |
| Nausea/vomiting | 72 ( 36.0 ) |
| Diarrhea | 38 ( 19.0 ) |
| Swelling | 14 ( 7.0 ) |
| Itching | 12 ( 6.0 ) |
| Rash | 10 ( 5.0 ) |
| Skin Lesions | 9 ( 4.5 ) |
| Unusual Bleeding | 0 ( 0 ) |
| Other | 2 ( 1.0 ) |
