# Supplementary material for: An Observational Study of Sepsis in Takeo Province Cambodia: An in-depth examination of pathogens causing severe infections
Source: PLoS Negl Trop Dis. 2020 Aug 17;14(8):e0008381. doi: 10.1371/journal.pntd.0008381 (PMC7430706; doi:10.1371/journal.pntd.0008381)
Supplement: S3 Table — (DOCX) [file pntd.0008381.s007.docx]

**S3 Table. Antimicrobial sensitivity test profiles of blood and sputum isolates.**

**% Susceptible**

|  |  | n= | IPM | C | AMP | AMC | CRO | CAZ | TMP/  SMZ | CIP | CN | AK | KZ | VA | MPM |
| --- | --- | --- | --- | --- | --- | --- | --- | --- | --- | --- | --- | --- | --- | --- | --- |
| **Blood Isolates** | | 17 |  |  |  |  |  |  |  |  |  |  |  |  |  |
| Escherichia coli | | 5 | 100  (5/5) | 67  (2/3) | 20  (1/5) | 40  (2/5) | 20  (1/5) | 40  (2/5) | 0  (0/5) | 0  (0/5) | 20  (1/5) | 100  (3/3) | 20 (1/5) | - | 100  (5/5) |
| Burkholderia pseudomallei | | 8 | 100  (8/8) | - | - | 100  (8/8) | - | 100  (8/8) | 50  (2/4) | - | 0  (0/8) | - | - | - | - |
| Klebsiella  pneumoniae | | 1 | 100 | 100 | 0 | 0 | 0 | 0 | 0 | 0 | 0 | 100 | 0 | - | 100 |
| Salmonella  typhi | | 1 | 100 | 100 | 100 | 100 | - | - | 100 | 0 | - | - | - | - | - |
| Proteus  mirabilis | | 1 | 100 | - | 0 | 100 | 100 | 100 | 0 | 0 | 0 | 0 | 0 | - | 100 |
| Streptococcus  suis* | | 1 |  |  | - | - | 100 | - | - | - | - | - | - | 100 | - |
|  | | **n=** | **IPM** | **C** | **AMP** | **AMC** | **CRO** | **CAZ** | **TMP/**  **SMZ** | **CIP** | **CN** | **AK** | **KZ** | **VA** | **MPM** |
| **Respiratory Isolates** | | 22 |  |  |  |  |  |  |  |  |  |  |  |  |  |
| Pseudomonas aeruginosa | | 2 |  | - | - | - | - | - | - | 100  (2/2) | 100  (2/2) | 100  (2/2) | - | - | - |
| Enterobacter sp. | | 1 | 100 | 0 | 0 | 100 | 0 | 100 | - | 0 | 0 | 100 | 0 | - | 100 |
| Klebsiella pneumoniae | | 7 | 100  (5/5) | 50  (1/2) | 0  (0/7) | 71 (5/7) | 71  (5/7) | 60  (3/5) | 86  (6/7) | 86  (6/7) | 86  (6/7) | 100  (3/3) | 57  (4/7) | - | 100  (5/5) |
| Klebsiella ozaenae | | 1 | 0 | - | 0 | 0 | 0 | 0 | 100 | 0 | 0 | 100 | - | - | 0 |
| Burkholderia pseudomallei | | 4 | 100 | - | - | 100  (4/4) | - | 100  (4/4) | 67  (2/3) | 0  (0/3) | 0  (0/3) | - | - | - | - |
| Haemophilus influenzae | | 2 | - | - | 0  (0/2) | 100  (1/1) | 100  (1/1) | - | 100  (1/1) | - | - | - | - | - | - |
| Staphyloccocus aureus | | 2 |  | - | - | - | - | - | 100  (1/1) | 100  (1/1) | - | - | - | 100  (1/1) | - |
| **CSF Isolate** | |  |  |  |  |  |  |  |  |  |  |  |  |  |  |
| Streptococcus suis | | 1 |  |  |  |  | 100 |  |  |  |  |  |  | 100 |  |

Abbreviations: AMC, Amoxicillin/Clavulanic Acid; AMP, Ampicillin; AK, Amikacin; KZ, Cephazolin; CAZ, Ceftazidime; CRO, Ceftriaxone; C, Chloramphenicol; CIP, Ciprofloxacin; OB, Cloxacillin; CN, Gentamicin; IPM, Imipenem; MPM, Meropenem; TMP/SMZ, Trimethoprim/Sulfamethoxazole; VA, Vancomycin; Coag-neg, Coagulase Negative; Those not tested are indicated with a dash (-). * indicates this patient was also culture positive for *Streptococcus suis* in wound pus.
